# Roux-en-Y gastric bypass surgery of morbidly obese patients induces swift and persistent changes of the individual gut microbiota

**DOI:** 10.1186/s13073-016-0312-1

**Published:** 2016-06-15

**Authors:** Albert Palleja, Alireza Kashani, Kristine H. Allin, Trine Nielsen, Chenchen Zhang, Yin Li, Thorsten Brach, Suisha Liang, Qiang Feng, Nils Bruun Jørgensen, Kirstine N. Bojsen-Møller, Carsten Dirksen, Kristoffer S. Burgdorf, Jens J. Holst, Sten Madsbad, Jun Wang, Oluf Pedersen, Torben Hansen, Manimozhiyan Arumugam

**Affiliations:** The Novo Nordisk Foundation Center for Basic Metabolic Research, Faculty of Health and Medical Sciences, University of Copenhagen, 2200 Copenhagen, Denmark; BGI-Shenzhen, Shenzhen, 518083 China; Department of Endocrinology, Hvidovre Hospital, 2650 Hvidovre, Denmark; Department of Biomedical Sciences, Faculty of Health Sciences, University of Copenhagen, 2200 Copenhagen, Denmark; Faculty of Health Sciences, University of Southern Denmark, 5000 Odense, Denmark; Danish Diabetes Academy, 5000 Odense, Denmark; iCarbonX, Shenzhen, 518083 China; Beijing Advanced Innovation Center for Food Nutrition and Human Health, College of Food Science and Nutritional Engineering, China Agricultural University, Beijing, 10083 China; Department of Biology, University of Copenhagen, 2200 Copenhagen, Denmark

## Abstract

**Background:**

Roux-en-Y gastric bypass (RYGB) is an effective means to achieve sustained weight loss for morbidly obese individuals. Besides rapid weight reduction, patients achieve major improvements of insulin sensitivity and glucose homeostasis. Dysbiosis of gut microbiota has been associated with obesity and some of its co-morbidities, like type 2 diabetes, and major changes of gut microbial communities have been hypothesized to mediate part of the beneficial metabolic effects observed after RYGB. Here we describe changes in gut microbial taxonomic composition and functional potential following RYGB.

**Methods:**

We recruited 13 morbidly obese patients who underwent RYGB, carefully phenotyped them, and had their gut microbiomes quantified before (n = 13) and 3 months (n = 12) and 12 months (n = 8) after RYGB. Following shotgun metagenomic sequencing of the fecal microbial DNA purified from stools, we characterized the gut microbial composition at species and gene levels followed by functional annotation.

**Results:**

In parallel with the weight loss and metabolic improvements, gut microbial diversity increased within the first 3 months after RYGB and remained high 1 year later. RYGB led to altered relative abundances of 31 species (*P* < 0.05, q < 0.15) within the first 3 months, including those of *Escherichia coli*, *Klebsiella pneumoniae*, *Veillonella* spp., *Streptococcus* spp., *Alistipes* spp., and *Akkermansia muciniphila.* Sixteen of these species maintained their altered relative abundances during the following 9 months. Interestingly, *Faecalibacterium prausnitzii* was the only species that decreased in relative abundance. Fifty-three microbial functional modules increased their relative abundance between baseline and 3 months (*P* < 0.05, q < 0.17). These functional changes included increased potential (i) to assimilate multiple energy sources using transporters and phosphotransferase systems, (ii) to use aerobic respiration, (iii) to shift from protein degradation to putrefaction, and (iv) to use amino acids and fatty acids as energy sources.

**Conclusions:**

Within 3 months after morbidly obese individuals had undergone RYGB, their gut microbiota featured an increased diversity, an altered composition, an increased potential for oxygen tolerance, and an increased potential for microbial utilization of macro- and micro-nutrients. These changes were maintained for the first year post-RYGB.

**Trial registration:**

Current controlled trials (ID NCT00810823, NCT01579981, and NCT01993511).

**Electronic supplementary material:**

The online version of this article (doi:10.1186/s13073-016-0312-1) contains supplementary material, which is available to authorized users.

## Background

Obesity affects millions of people worldwide and its prevalence is increasing at a pandemic level. The causes of this complex disease include genetic predisposition, epigenetic changes, lifestyle habits, and a range of environmental factors [1–3]. As obesity is the main risk factor for the development of life-threatening comorbidities such as type 2 diabetes (T2D) and coronary heart disease [4], new strategies for the prevention and treatment of obesity are urgently needed. Roux-en-Y gastric bypass (RYGB) surgery is currently the most effective treatment for severely obese individuals as it induces rapid and sustained weight loss and a significant improvement in glucose metabolism and insulin sensitivity [5–7]. Post-surgery effects also include improvements in inflammatory markers [8] and reduction of adiposity [9, 10]. Although the exact mechanisms underlying these beneficial effects of RYGB are not yet fully understood, a few factors have been suggested to play a key role: decrease in appetite and meal size [11], change in food preferences, anatomical rearrangement of the gut, significant changes in the secretion of satiety-related intestinal peptides such as glucagon-like peptide-1 (GLP-1) [12–14], and a shift in bile acid metabolism [15].

The gut microbiota has been hypothesized as a factor linking food intake to obesity, metabolic alterations, and intestinal inflammation [5, 16]. Some microbes produce pro-inflammatory molecules, such as lipopolysaccharides, which may affect host metabolism through proteins produced by the host to mediate the immune response [16, 17]. Moreover, obesity has been associated with altered gut microbiota composition [18–20], reduced microbial diversity [21], and reduced gene richness [22]. Dietary weight loss interventions in humans have resulted in an increase in microbial gene richness and a shift from obese to lean microbial compositions [19, 23]. Also, diets with different proportions of fat, carbohydrates, and proteins have been associated with changes in the microbiota composition in humans [24, 25] and rodents [26–28]. Finally, the gut microbes contribute to regulation of energy homeostasis and fat storage [16, 22, 29–31].

As RYGB leads to metabolic improvements, and metabolic changes are associated with gut microbial changes, an important open question is whether specific changes in the gut microbiota occur following RYGB. Previous studies investigating changes in the gut microbiota after bariatric surgery have observed increased microbial diversity and altered microbial composition, primarily an increased relative abundance of the phylum *Proteobacteria* in both humans [32–35] and rodents [36, 37]. Studies also suggest that these microbial changes may be independent of weight loss or caloric restriction, maintained up to 9 years after surgery, and are not confounded by pre-surgery body mass index (BMI) [10, 37]. Furthermore, colonization of germ-free mice with fecal material from RYGB-operated mice caused weight loss and reduced adiposity, providing evidence that RYGB-associated gut microbiota can improve host metabolism [10, 37]. None of the studies has followed the same subjects for more than 6 months, however, and it is not clear whether gut microbial changes occur within a short period after RYGB or gradually over a longer period.

Here we present a longitudinal shotgun-sequencing-based metagenomics study of 13 morbidly obese patients examined before (baseline) and 3 months (n = 12) and 1 year after RYGB (n = 8). The aim of the study was to investigate short- and long-term changes in gut microbial composition and functional potential following RYGB-induced intestinal rearrangement and associated changes in body weight and metabolism.

## Methods

### Study participants

Study participants were recruited at Hvidovre Hospital, Denmark as a part of the bariatric surgery program. All patients had accomplished a preoperative 8 % diet-induced total body weight loss before inclusion and met the Danish criteria for bariatric surgery: (i) >20 years old and (ii) either BMI >40 kg/m^2^ or BMI >35 kg/m^2^ with T2D/hypertension. Fecal samples were collected as a part of three larger studies investigating the effects of RYGB on glucose metabolism [14, 38, 39]. In total, 13 patients (five men and eight women) with available fecal samples at baseline were included in the current study (Additional file 1: Figure S1). Of these, seven patients had T2D pre-surgery, one had impaired glucose tolerance, and five had verified normal glucose tolerance. All patients received injections of vitamin B12 as well as dietary supplements post-surgery in the form of calcium, vitamin D, and multivitamin tablets.

### Anthropometric and biochemical measurements

Participants were examined before and 3 months and 1 year after RYGB. On the day of study, participants were examined after a 12-h overnight fast and subjected to a liquid meal test as reported [14, 38, 39]. Blood samples were drawn in the fasting state and at eight time points after meal intake (−10, −5, 0, 15, 30, 45, 60, 90, 120, 180, and 240 minutes relative to meal start). Anthropometrics were measured and plasma (p) glucose, serum (s) insulin, p-GLP-1, and glycated hemoglobin A1c (HbA1c) were analyzed as described [14, 38, 39]. The area under the curve (AUC) for p-glucose and p-GLP-1 was calculated using the trapezoidal method.

### Stool sample collection, DNA extraction, and metagenomic sequencing

Stool samples were collected before RYGB (n = 13) as well as 3 months (n = 12) and 1 year (n = 8) after the surgery (Additional file 1: Figure S1). Patients collected fresh stool samples at home that were immediately frozen in their home freezer at −20 °C. Frozen samples were delivered to the hospital within 2 days using insulating polystyrene foam containers and were stored at −80 °C until DNA extraction.

Microbial DNA was extracted from 200 mg of frozen stool using the International Human Microbiome Standards (IHMS) standard operating procedure 07 V2 (http://www.microbiome-standards.org/index.php?id=254). The concentration and quality of the extracted DNA were estimated using a Qubit Fluorometer (from Thermo Scientific) and agarose gel electrophoresis. Whole genome shotgun sequencing was performed on the 33 fecal samples using the Illumina HiSeq 2000 platform and paired-end sequencing method (2 × 100 bp). We generated, on average, 76 million reads per sample. Reads were quality controlled, accepting only reads with a quality trimming cutoff of 20 and a minimum length of 30 bp [40]. Contaminating human DNA sequences were removed by screening them against the human genome (hg19). Sample information and read quality control summary statistics are provided in Additional file 2: Table S1.

### Taxonomic profiling of fecal metagenomes

Taxonomic abundance profiles were generated by MOCAT software [40] by aligning screened high-quality reads (alignment length cutoff 30 and minimum 97 % sequence identity for the option “screen”) to a database consisting of ten universal single-copy marker genes extracted from 3496 NCBI reference genomes and 263 metagenomes [41]. We obtained abundances for 477 species-level metagenomic operational taxonomic units (mOTUs). Taxa were merged if their NCBI species annotation were the same (e.g., multiple mOTUs were annotated as *Faecalibacterium prausnitzii* and *Fusobacterium nucleatum*).

### Functional annotation and functional profiling of fecal metagenomes

An average of 77 % high-quality reads per sample were mapped to the recently published 9.9 million gene catalog established from cohorts of three different continents [42]. From this catalog we used the 42.1 % genes annotated with the Kyoto Encyclopedia of Genes and Genomes (KEGG) orthology [43, 44] to obtain KEGG orthologous group profiles. Abundances were then calculated for KEGG modules and pathways by summing the abundances for each KEGG orthologous group that belonged to the same module or pathway, respectively.

### Relative abundance calculation and microbial feature selection

The abundances quantified by MOCAT at the species level were transformed to relative abundances by dividing them by the total abundance per sample, including the high-quality reads that could not be annotated to any reference genome or metagenome. The species relative abundances were summarized to phylum levels based on the NCBI taxonomy by summing the relative abundances of all the members belonging to the same phylum. We removed low-abundance microbial features as follows. Firstly, we removed microbial features (taxa and functional units) that were present in <10 % of all the samples. Secondly, we removed taxa and functional units (KEGG modules or pathways) whose average relative abundance across all the samples was lower than 0.01 and 0.001, respectively. This filtering resulted in nine phyla, 105 mOTU species, 266 KEGG modules, and 212 KEGG pathways for the subsequent differential analyses. Functional units that were not prokaryotic are not discussed in the “Results” section. When calculating fold changes, we added a pseudo-count to the relative abundances, which was the lowest relative abundance observed for the entire cohort.

### Addressing compositional effects

Metagenomic studies of microbial communities sample a fraction of the total genomic content (sampling depth), which is then sequenced at a certain sequencing depth. Both sampling depth and sequencing depth can vary by several orders of magnitude between samples. As absolute microbial counts (abundances) are normally not known and measurements depend on sampling and sequencing depths, community compositions are represented using relative abundances [45]. Since relative abundances are constrained (they must sum to 1 in a given community), they are susceptible to compositional effects where an increase in relative abundance of one component leads to a compositional decrease in the relative abundance of other components. Differential analysis based on relative abundances thus needs careful interpretation as compositional effects can introduce spurious differences in relative abundances while the absolute abundances are not different. We developed a simple method that enabled us to evaluate if our results were biologically real or a consequence of studying compositional data (relative abundances). The method is a simple algorithm that tests if the fold change of each taxon varies between time points when we exclude each of the other taxa from the relative abundance table. The algorithm proceeds, first, by leaving one taxon out of the relative abundances table. Second, it renormalizes the table by dividing the relative abundances by the total sum of relative abundance to make all the taxa relative abundances sum to 1 again. Lastly, it calculates the fold change (log2) between time points and performs a Wilcoxon signed-rank test for each taxon. We repeated this process for all the taxa considered and evaluated whether our results (fold change of microbes) were spurious or not using the least significant *P* value calculated for each taxon. We have published the R source code for this algorithm at GitHub (https://github.com/apalleja/compositionality_test/).

### Statistical analysis

The anthropometric and clinical measures have been reported previously [14, 38], but here we present these data as a function of time. To have a better overview of how they globally change during the study time frame, we projected their values at the three time points into the principal component analysis (PCA) space. We also measured their change normalized by months (changes between baseline and 3 months divided by 3 and between 3 months and 1 year divided by 9). Species and gene richness and the Shannon diversity index were evaluated to estimate the microbial diversity before and after RYGB.

PCA was also performed on the log10 transformed relative abundances of the mOTU species. Permutational multivariate analysis of variance (PERMANOVA) was used to assess the effects of the surgery (before/after), glycemic status before surgery (normal glucose tolerant or T2D), usage of metformin before surgery (five of seven T2D patients had taken metformin before surgery), BMI, waist/hip ratio, and postprandial p-GLP-1. We did not include in the analysis fasting p-glucose, fasting p-insulin, HbA1c, and postprandial p-insulin and p-glucose as they are collinear with glycemic status. We performed the analysis using the function “adonis” in the “vegan” package in R. A distance matrix was obtained by calculating Canberra distances among samples based on the relative abundances of mOTU species and the permuted *P* value was obtained by 10,000 permutations, as performed in a previous study [46]. We controlled for multiple testing using Benjamini–Hochberg (BH) false discovery rate (FDR).

We used a non-parametric statistical test to show which microbial features (taxa and functional units) changed their abundance significantly between time points. Since this is a longitudinal study and samples are therefore not independent, we used a two-sided Wilcoxon signed-rank test, which accounts for paired samples. The *P* value distribution for each statistical test was analyzed to examine how our test performed across all hypotheses. The proportion of null hypothesis on these *P* value distributions was estimated by the “q value” method (http://github.com/jdstorey/qvalue) [47], which has been previously used in gut microbiome analysis [29, 48, 49] (these are reported in Additional file 1: Figures S5–S10). To correct for multiple testing, we also calculated q values using the BH FDR. For each test we reported unadjusted *P* values and their corresponding BH FDR q values (q). We used a consistent unadjusted *P* value cutoff of 0.05. As different feature types exhibited different distributions of *P* values, the same unadjusted *P* value cutoff leads to different estimates of FDRs and adjusted q values for different features, ranging from q < 0.04 to q < 0.22. Our study was underpowered (n = 13, n = 12, n = 8 in three time points) to test hundreds of features (105 microbial species and 266 microbial functions) with stringent cutoffs (such as q < 0.05). Therefore, to avoid missing possible effects of RYGB in low-abundance microbial species and functions, we allowed lenient FDRs corresponding to *P* < 0.05, namely q < 0.08, q < 0.15, q < 0.16, q < 0.17, and q < 0.22. However, to ensure that results are interpreted with caution, we explicitly reported the upper bounds for number of false positives when the FDR was above 10 %. We did discard microbial changes in three cases when *P* < 0.05 corresponded to extremely high FDRs, namely q > 0.86, q > 0.91, and q > 0.99. For completeness we also report the q values from the Storey “q value” method in Additional file 1: Tables S3 and S4. They are generally quite similar to the BH FDR q values. All the data analysis and statistical testing were performed with R (version 3.2.0; http://www.R-project.org/).

## Results and discussion

### Gut microbial diversity increases after RYGB in parallel with metabolic improvements

**Fig. 1:**
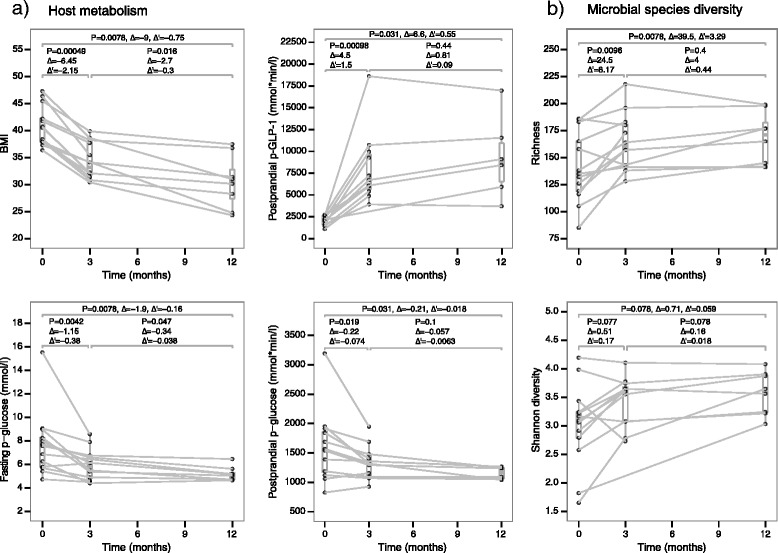
Metabolic and microbial diversity improvements during a 1-year period after RYGB. *Box plots* represent features measured at the three different time points. *Lines* connect the measures from the same subject. For each pairwise comparison between time points, the *P* value of the Wilcoxon signed-rank test (*P*), the difference between the medians (Δ), and difference between medians normalized by time difference (Δ′) are denoted. **a** Host metabolism improvements. Postprandial glucose and GLP-1 levels were calculated as area under the curve during a standardized meal test. **b** Microbial species diversity improvements

We have previously reported that, 3 months after RYGB surgery, BMI, fasting p-glucose, fasting s-insulin, and HbA1c significantly decreased, while postprandial p-GLP-1 secretion significantly increased in the subjects studied here [14, 38, 39]. The projection of all the phenotypic data considered in our study on the principal component space showed an overall change in anthropometric and clinical markers after RYGB (Additional file 1: Figure S2). We investigated whether the metabolic improvements and gut microbial changes occurred within short-term (within 3 months) or long-term following RYGB using samples collected 3 months and 1 year after RYGB. Additionally, to study the role of gut microbiota in relation to the metabolic improvements, we chose to investigate BMI, fasting p-glucose, postprandial p-glucose, and postprandial p-GLP-1 as indicators of health status after RYGB. BMI, fasting p-glucose, and postprandial p-GLP-1 differed between baseline and 3 months (Additional file 1: Figure S3; Wilcoxon signed-rank test; *P* = 0.00049, *P* = 0.0042, and *P* = 0.00098, respectively). Only BMI and fasting p-glucose differed between 3 months and 1 year after RYGB (Additional file 1: Figure S3; Wilcoxon signed-rank test; *P* = 0.016 and *P* = 0.047, respectively). However, when we normalized the changes by the number of months within each time interval, we observed that the shift towards a healthier metabolism occurred mainly within the first 3 months after RYGB (Fig. 1a). Though these improvements were maintained during the following 9-month period, the rate of improvement was markedly lower.

We estimated gut microbial species compositions using the species-level mOTU approach, based on single-copy phylogenetic marker genes [41]. As previous studies have shown a positive association between a healthy metabolic state and increased microbial diversity and gene richness [22, 23], we first estimated the microbial alpha-diversity using three measures: species richness, species Shannon index, and gene richness. Compared with baseline, the Shannon index at the species level showed a weak tendency to increase 3 months and 1 year after RYGB (Additional file 1: Figure S4a; Wilcoxon signed-rank test; *P* = 0.077 and *P* = 0.15, respectively). While species richness was higher 3 months after RYGB and this higher richness was maintained at 1 year (Wilcoxon signed-rank test; *P* = 0.0096 and *P* = 0.0078, respectively; Additional file 1: Figure S4b), gene richness exhibited a tendency to increase only after 1 year (Wilcoxon signed-rank test; *P* = 0.078; Additional file 1: Figure S4c). The discrepancy between species richness and gene richness could be due to lack of power when using n = 12 samples. When we normalized the changes in species richness and Shannon index by the number of months (Fig. 1b), we observed that most changes occurred within the first 3 months and were merely maintained during the last 9 months. Thus, the microbial diversity improvements mirrored the trends of metabolic improvements.

### Persistent changes in the gut microbial composition induced by RYGB

**Fig. 2:**
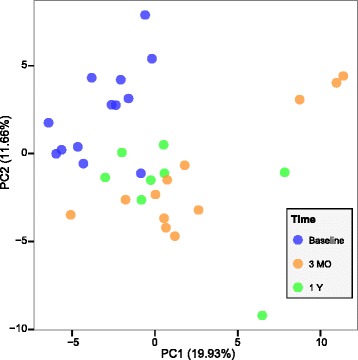
Gut microbial community differences induced by RYGB. Principal component analysis based on log transformed mOTU species abundances shows a clear separation between pre-RYGB and post-RYGB fecal samples. The variation explained by each component is shown on its axis. *MO* months, *Y* year

We visualized the changes in overall gut microbial species composition induced by RYGB using a principal component analysis of the log-transformed relative abundances (Fig. 2), which showed a clear separation between baseline samples and those after RYGB. Such separation could not be observed between 3-month and 1-year samples, suggesting that most of the changes in microbial composition occurred within 3 months and those changes were maintained up to 1 year. RYGB induces physiological and metabolic changes in the subjects, which may also be contributing to the changes in the microbiome composition. In order to evaluate this, we performed a permutational analysis of variance (PERMANOVA) test to quantify the variance explained by RYGB as well as other physiological and metabolic parameters. RYGB surgery, T2D status (before surgery), metformin usage (before surgery), p-GLP-1 levels (at each time point), and BMI (at each time point) explained the variation in species composition (*P* < 0.05; q < 0.06; Additional file 2: Table S2). As we included both normal glucose tolerant and T2D patients, our PERMANOVA results suggest that RYGB alters the gut microbial composition in both groups. Our finding on the effect of T2D status agrees with previous reports on altered gut microbial composition in T2D patients [29, 30]. It is interesting to note that baseline T2D status had an effect on gut microbial composition up to 1 year after RYGB. At baseline, most T2D patients (five out of seven) in the cohort were taking metformin, which has recently been reported to alter gut microbial composition and confound the gut microbial signatures associated with T2D [46]. Therefore, we cannot distinguish the microbial changes due to T2D status from microbial changes induced by taking metformin. Previous studies have hypothesized that GLP-1 secretion can be stimulated by bacterial metabolites such as short chain fatty acids through GPR41/43-dependent mechanisms [50], which could explain the association between p-GLP-1 levels and gut microbial composition. Finally, PERMANOVA results suggest that BMI could explain the variation in gut microbial composition but to a lesser extent than the surgery. Previous studies have reported that the gut microbial changes identified by them were not confounded by BMI [10, 37]. Our results do not contradict their conclusions but suggest that, in our cohort, BMI can explain some additional variation in gut microbial composition beyond what is explained by RYGB.

We next investigated compositional changes in individual taxa following RYGB using Wilcoxon signed-rank tests on relative abundances. Verifying taxonomic changes using relative abundance is susceptible to compositional effects, where an isolated increase in absolute abundance of just one taxon will lead to a dissipated decrease in relative abundance of all other taxa as the relative abundances must always sum to 1 [45]. Although there is an ongoing discussion about how to differentiate compositionality-induced changes from real changes [45, 51–53], this is not commonly addressed in microbiome studies. We developed a procedure to assess whether compositionality had influenced our results. When a taxon exhibited a significant difference in relative abundance between two time points, we verified whether this difference was a compositional effect due to a difference in another taxon. We tested if the former would still exhibit a difference if the latter was never observed in any of the samples. By systematically repeating this procedure for all other taxa and evaluating the least significant *P* value, we could discard spurious differences arising due to compositional effect (see “Methods” for details).

**Fig. 3:**
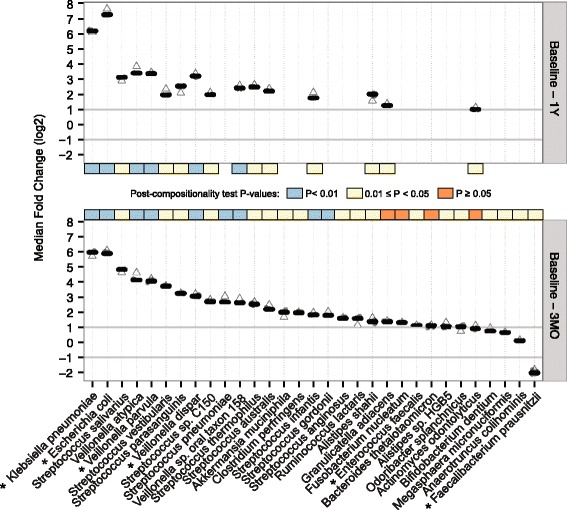
Changes in individual gut microbial species following RYGB. Median fold changes in relative abundances of 31 mOTU species that changed between baseline and 3 months (*3MO*, *bottom panel*), and 16 among these that changed between baseline and 1 year (*1Y*, *top panel*) after RYGB. For each bacterial species, the cloud of *circles* represents all fold changes calculated when excluding one other species from the abundance table. The *horizontal grey lines* at −1 and 1 mark when the microbes halved or doubled their relative abundance. Exclusion of *Prevotella copri* substantially altered the fold change for many species and the corresponding fold change is denoted as an *empty triangle*. The *colored band* in each panel shows the statistical significance of Wilcoxon signed-rank tests after our compositionality test. *Asterisks* mark species that have already been reported in previous studies

At the phylum level, compared with baseline, *Proteobacteria* and *Fusobacteria* showed an increase in relative abundance 3 months after RYGB (Wilcoxon signed-rank test, *P* < 0.05, q < 0.04; Additional file 2: Table S3). The same two phyla exhibited increased abundance levels after 1 year compared with baseline (*P* < 0.05, q < 0.08; Additional file 2: Table S4) and we did not observe any significant phylum level changes between 3 months and 1 year (q > 0.91). At the species level, 31 species changed their relative abundance within the first 3 months (Wilcoxon signed-rank test; *P* < 0.05, q < 0.15, suggesting that up to five species could be false positives; Fig. 3; Additional file 1: Figure S5; Additional file 2: Table S3). Nineteen species changed between baseline and 1 year (*P* < 0.05, q < 0.22, suggesting that up to five could be false positives; Fig. 3; Additional file 1: Figures S6; Additional file 2: Table S4), including 16 of the 31 species that changed within the first 3 months. However, we did not observe significant changes in species abundances when comparing the gut microbiota composition at 3 months and 1 year after RYGB (Wilcoxon signed-rank test; q > 0.99; Additional file 1: Figure S7), which provides further evidence that the remodeling of the microbial community occurred mainly within the first 3 months after the surgery. Figure 3 shows the RYGB-associated fold changes for these 31 differentially abundant species. When we performed the test for compositional effect, only four of these species lost their significance (*P* > 0.05) when another species was considered absent (Fig. 3), suggesting that most taxonomic changes reported here are not affected by compositionality. Most of the 31 species, including two affected by compositionality (*Actinomyces odontolyticus* and *F. nucleatum*), exhibited a marked difference in their fold change when *Prevotella copri* was considered absent. The genus *Prevotella* is the main driver of the *Prevotella* enterotype [54] and exhibits a bimodal distribution, with high relative abundance in some individuals and a low relative abundance in others [55], which explains why simulating the absence of *P. copri* leads to marked changes in the relative abundance of other species.

*Escherichia coli* and *Klebsiella pneumoniae* showed a dramatic increase after RYGB (Fig. 3)*.* Previous studies have reported the increase of *E. coli* after RYGB and have pointed to its higher efficiency to harvest energy during host starvation status, giving them an advantage in the post-RYGB starvation-like condition during the first months after the surgery [34]. Ten species belonging to the genus *Streptococcus*, four from *Veillonella*, two from *Alistipes*, *Bifidobacterium dentium*, *Enterococcus faecalis*, *F. nucleatum*, and *Akkermansia muciniphila* also increased their abundance after RYGB (Fig. 3). Our results agree with previous findings that *A. muciniphila*, a mucin-degrading bacterium, is associated with reduction in adiposity, inflammation, glucose intolerance, and body fat mass [56]. The increase in aero-tolerant *Proteobacteria*, including *Streptococcus* spp., *E. coli*, *K. pneumoniae*, and *E. faecalis*, might result from a higher presence of oxygen in distal parts of the gut due to the anatomical rearrangements as reported previously [35, 57]. Changes in pH after RYGB may also affect these aero-tolerant anaerobic microbes by inducing changes in the redox potential of the gut [58]. Furthermore, a decrease in acid secretions due to the reduced size of the stomach could make the gastric barrier less stringent for oral microbiota such as *Streptococcus* spp., together with *F. nucleatum*, *B. dentium* and a few *Veillonella* spp., which are metabolically dependent on *Streptococcus* spp. in oral biofilms [59]*.* The only species that decreased after RYGB in our study was the butyrate-producing *F. prausnitzii*, which is surprising as it has been associated with beneficial effects on host metabolism and negatively correlated with inflammation markers [60]. A previous study using metagenomic sequencing has also reported a post-RYGB decrease of *F. prausnitzii* in six obese T2D patients [35]. On the contrary, another study using quantitative PCR reported that, in obese T2D patients, *F. prausnitzii* showed a trend to increase 3 months after RYGB and stayed at the increased level 6 months after RYGB [33]. The latter study also reported that, in obese non-diabetic individuals, *F. prausnitzii* decreased significantly 3 months after RYGB and returned back to basal levels 6 months after RYGB. Here we observed that *F. prausnitzii* decreases following RYGB for most of the diabetic and non-diabetic patients. Taken together, our study has reproduced six previously observed species-level changes in obese individuals after RYGB (*E. coli*, *K. pneumoniae*, *Veillonella dispar*, *Veillonella parvula*, *E. faecalis*, and *F. prausnitzii*). Previous studies have also reported changes in the relative abundance of *Acinetobacter* spp., *Citrobacter* spp., *Clostridium* spp., *Enterobacter* spp., *Pseudomonas* spp., *Shigella* spp., *Staphylococcus* spp., *Vibrio* spp., and *Yersinia* spp. [10, 32, 35], which we could not verify in our study (see Additional file 2: Table S5 for the full list of species). We also observed an increase in *Alistipes* spp., *Streptococcus* spp., two other *Veillonella* spp., and *A. muciniphila* that are unique to this study (see Additional file 2: Table S5 for the full list of species).

### Altered microbial functions after RYGB

**Fig. 4:**
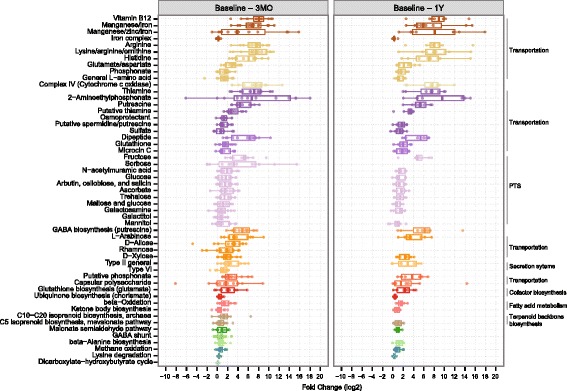
Microbial functional changes following RYGB. *Box plots* represent fold changes (log2) in the 53 KEGG modules that increased in relative abundance between baseline and 3 months (*3MO*, *left panel*) and 44 among these that increased between baseline and 1 year (*1Y*, *right panel*) after RYGB. The different KEGG functional categories are represented by different *colors* and grouped together when possible with corresponding labels at the *right side* of the plot. *PTS* phosphotransferase systems, *GABA* gamma-aminobutyric acid

To characterize the changes in functional potential of microbes to adapt to the gut rearrangement after RYGB, we estimated the relative abundances for KEGG modules and pathways in each sample. We found 62 KEGG modules that changed in relative abundance between baseline and 3 months after RYGB (Wilcoxon signed-rank test, *P* < 0.05, q < 0.17, suggesting that up to 11 modules could be false positives; Additional file 1: Figure S8; Additional file 2: Table S3), and 63 KEGG modules that changed between baseline and 1 year (Wilcoxon signed-rank test, *P* < 0.05, q < 0.16, suggesting that up to ten could be false positives; Additional file 1: Figure S9; Additional file 2: Table S4), while we did not observe significant changes between 3 months and 1 year (q > 0.86; Additional file 1: Figure S10). Most of these changes (53 out of 62 in the former and 56 out of 63 in the latter) reflected an increase in relative abundance over time (Fig. 4), which may reflect the increased species richness after RYGB. Of the 53 modules that increased their abundance within the first 3 months, 44 sustained it for a year. Thus, the functional changes mirrored the taxonomic changes, where most changes had occurred during the first 3 months and were merely maintained during the following 9 months.

Among the KEGG modules that increased their abundance after RYGB, we observed several nutrient transport systems (Fig. 4). Morbidly obese patients who have undergone bariatric surgery have a smaller stomach pouch and their food intake skips the duodenum, which causes malabsorption of essential vitamins, minerals, and drugs [61]. To compensate for this malnutrition, patients follow a diet rich in proteins and take calcium, iron, and multivitamin supplements (see “Methods”). We observed an increased potential for microbial transport systems of thiamine, vitamin B12, manganese, iron, and zinc (Fig. 4), which could reflect the increased availability of these compounds that are also essential for microbes. Transport systems of phosphonates were also increased after RYGB. Some bacteria such as *E. coli* and *Klebsiella* spp. can utilize these compounds as an alternative source of phosphorus by breaking their C–P bonds [62].

There was also an increased abundance of transport systems for monosaccharides such as D-xylose, rhamnose, D-allose, and L-arabinose after RYGB. In *E. coli*, the presence of L-arabinose in the absence of glucose dynamically activates the operon that drives the catabolism of arabinose [63]. Arabinose is present in rice, wheat, beans, oats, or plant polysaccharides. Thus, our finding may reflect changes in diet, for example, a shift in food preferences towards lower-calorie-dense foods as reported after RYGB [64–66].

Other transport systems that increased in abundance after RYGB are the phosphotransferase systems (PTS; Fig. 4), which are only found in bacteria. PTS catalyze the transport and phosphorylation of numerous monosaccharides, disaccharides, amino sugars, polyols, and other sugar derivatives into the bacterial cell. Their increase could be attributed to an increased ability of microbes to assimilate all available sugars to compensate for the reduced dietary intake. Figure 4 also shows the increased potential of amino acid uptake, suggesting the utilization of amino acids as a source of energy, and an increased potential for beta-oxidation of fatty acids, indicating the utilization of these fatty acids as a source of energy.

The KEGG module for cytochrome c oxidase complex and the module for prokaryotic biosynthesis of ubiquinone also increased in abundance after RYGB. The former is the last enzyme of the electron transport chain in both bacteria and eukaryotic mitochondria. Ubiquinone, known as coenzyme Q_10_, also plays a crucial role as an electron carrier in the electron transport chain. The increased relative abundance of these two modules together with the increase of facultative anaerobes such as *E. coli*, *K. pneumoniae*, *E. faecalis*, and *Streptococcus* spp. suggest a shift towards aerobic respiration among the facultative anaerobes to benefit from a higher presence of dissolved oxygen in the hindgut after RYGB [57]. Electron transport chains are major sites of premature electron leakage to oxygen, generating superoxide and potentially resulting in increased oxidative stress. Post-RYGB, we observed an increased abundance of a module encoding glutathione biosynthesis from glutamate and an increased abundance of transport systems of both glutamate and glutathione (Fig. 4). In bacteria, glutathione, in addition to its key role in maintaining the proper oxidation state of protein thiols, also protects the cell from oxidative and osmotic stress [67]. Thus, the increased capacity in glutathione biosynthesis and transport suggests that the gut microbes may be using glutathione to combat oxidative stress.

Intriguingly, following RYGB we observed an increase in abundance of two KEGG modules involved in putrescine transportation (Fig. 4). Although protein digestion is not impaired after RYGB [68], the increased potential for putrescine transportation might indicate a certain level of putrefaction in the colon, as other authors have hypothesized when studying the fecal metabolic profiles after RYGB in a non-obese rat model [36]. Fast pouch emptying and a delayed small intestinal transit time have been reported for RYGB patients [69]. Reduction of gastric acid secretion after surgery [70, 71], which plays a key role in protein digestion by activating proteolytic enzymes, might cause more incompletely digested proteins to reach the colon. Under these circumstances, a longer intestinal transit time could provide enough time for microbes to catabolize these proteins, resulting in the production of polyamines such as putrescine [36, 72], which is involved in key functions such as DNA and membrane stabilization but becomes toxic at high doses and can even produce carcinogenic nitrosamines [73]. Putrescine has been found in elevated concentrations in fecal samples post-RYGB in rats [36]. *Enterobacteriaceae* spp. such as *E. coli* or *Klebsiella* spp. can produce putrescine by decarboxylation of the amino acids ornithine and arginine [73], of which we also observed an increased transportation potential after RYGB (Fig. 4). The increased capacity to transport putrescine could also reflect the antioxidant and anti-inflammatory properties of this polyamine for microbes when oxidative stress increases [74–76]. Microbial processing of putrescine can produce gamma-aminobutyric acid (GABA) [77], which is an inhibitory neurotransmitter of the mammalian central nervous system and has been found increased in fecal samples after RYGB in a rat model [36]. This neurotransmitter is thought to stimulate the intestinal cells to release GLP-1 [78, 79]. Increased levels of GLP-1 observed after RYGB in our cohort (Fig. 1; Additional file 1: Figure S3) is consistent with this link. The increase in GLP-1 can, in turn, stimulate the biosynthesis of GABA via pancreatic beta-cells [80]. An increased capacity for GABA biosynthesis and GABA shunt (closed loop to produce and maintain the supply of GABA) pathways observed after RYGB (Fig. 4) provides further evidence for this metabolic path after RYGB.

## Conclusions

We recruited morbidly obese human patients undergoing RYGB, performed a longitudinal study of the effects of RYGB on gut microbiota, and compared the short-term effects (after 3 months) with the long-term effects (after 1 year). Our analyses showed an increased gut microbial diversity and an altered microbial composition in conjunction with the metabolic improvements seen after RYGB. Most of these changes occurred within the first 3 months and were maintained during the following 9 months. Although we cannot prove a causal role for gut microbial changes in relation to the metabolic improvements, a recent study has shown that transferring post-RYGB microbiota from humans to germ-free mice leads to fat mass regulation [10], suggesting that the altered microbiota could contribute to metabolic changes. Thus, our study opens up new possibilities for thorough characterization of gut microbial changes immediately following RYGB in order to verify their contribution to metabolic health improvement.

**Fig. 5:**
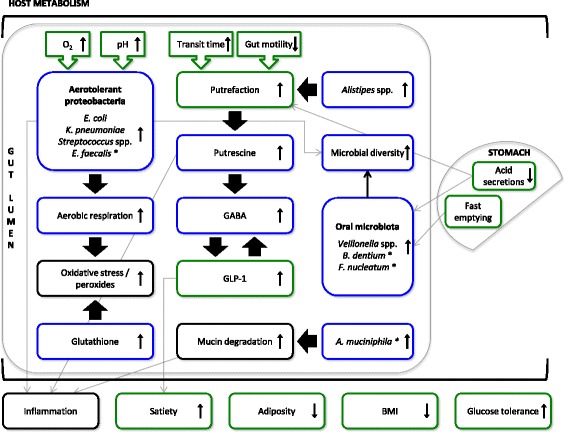
A model of gut microbial changes following RYGB. *Blue boxes* show inferred changes in microbial features (functional potential or taxonomic), while *green boxes* show the effects induced by RYGB either in the gut or in the host metabolism. *Black boxes* indicate hypotheses based on our data or other studies. *Arrows* connect shifts that are related. Since we did not measure inflammation markers we do not report an increase or decrease in inflammation, but we connect it to an observed change based on existing literature. All features shown here exhibited changes 3 months after RYGB and most maintained the changes up to 1 year after RYGB. *Asterisks* denote features that did not maintain the changes 1 year after RYGB

We also developed procedures to rule out spurious changes in microbial relative abundance due to compositional effects. Microbial changes observed here were RYGB-specific and different from changes due to weight loss interventions [26]. Our interpretation of data is in accordance with recent observations in mice [37]. Collectively the available evidence suggests that the microbial changes after RYGB are more driven by the intestinal rearrangement rather than weight loss. *Proteobacteria* and *Fusobacteria* increased their relative abundance and the butyrate-producer *F. prausnitzii* decreased after RYGB. The increase in abundance of aero-tolerant bacteria from the phylum *Proteobacteria*, together with the increased abundance of genes encoding key components in the electron transport chain, indicated adaptation to a higher presence of oxygen in the distal gut after RYGB, as previously reported [32, 35, 57] (Fig. 5). Increased abundance of various systems of transportation and uptake of vitamins, minerals, organic compounds, simple sugars, and amino acids could suggest an increased potential of microbes to assimilate essential compounds and all possible energy substrates as compensatory mechanisms to counteract reduced food intake after RYGB. We observed an increased microbial potential to transport putrescine, which could even contribute to a higher secretion of GLP-1 via GABA biosynthesis (Fig. 5). In future studies, it would also be relevant to measure the fecal putrescine content to assess the extent to which protein putrefaction occurs after RYGB and to relate this measure to any potential health risk that may be caused by this toxin.

By identifying swift and consistent changes that occurred within 3 months and were maintained for a year in morbidly obese humans, we have modeled the persistent gut microbial changes induced by RYGB (Fig. 5). Further studies characterizing such changes at a finer time scale immediately after surgery will shed more light on the dynamic adaptation of gut microbiota to RYGB and their role in metabolic improvements.

## Additional files

Additional file 1: Ten supporting figures. A figure caption for each is given within the file. (PDF 716 kb) Additional file 2: Five supporting tables. A table caption of each is given within the file. (XLSX 84 kb)

## Abbreviations

BH: Benjamini–Hochberg
BMI: body mass index
FDR: false discovery rate
GABA: gamma-aminobutyric acid
GLP-1: glucagon-like peptide-1
HbA1c: hemoglobin A1c
KEGG: Kyoto Encyclopedia of Genes and Genomes
mOTU: metagenomic operational taxonomic unit
p: plasma
PCA: principal component analysis
PERMANOVA: permutational multivariate analysis of variance
PTS: phosphotransferase systems
RYGB: Roux-en-Y gastric bypass
s: serum
T2D: type 2 diabetes
