# Supplementary material for: Roux-en-Y gastric bypass surgery of morbidly obese patients induces swift and persistent changes of the individual gut microbiota
Source: Genome Med. 2016 Jun 15;8:67. doi: 10.1186/s13073-016-0312-1 (PMC4908688; doi:10.1186/s13073-016-0312-1)
Supplement: Additional file 1: — Ten supporting figures. A figure caption for each is given within the file. (PDF 716 kb) [file 13073_2016_312_MOESM1_ESM.pdf]

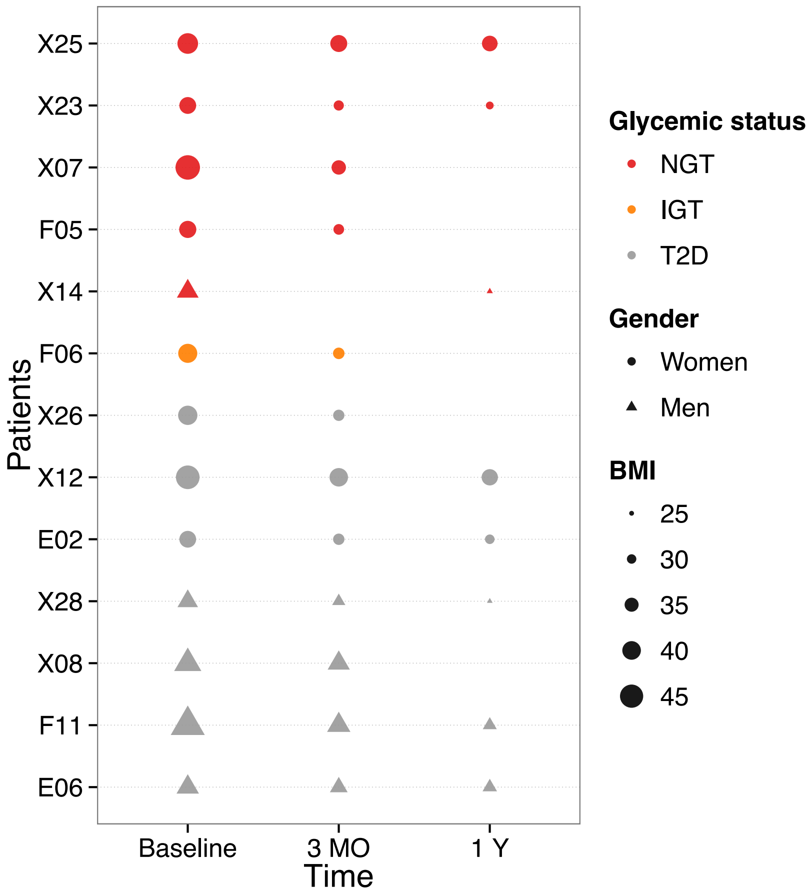

Figure S1. Patients overview. The presence or absence of a symbol represents whether we obtained or not a sample for the patient at the specific time point. As it is shown in the legend at right, the size of the symbol indicates the BMI of each patient, which decreases over time after RYGB.

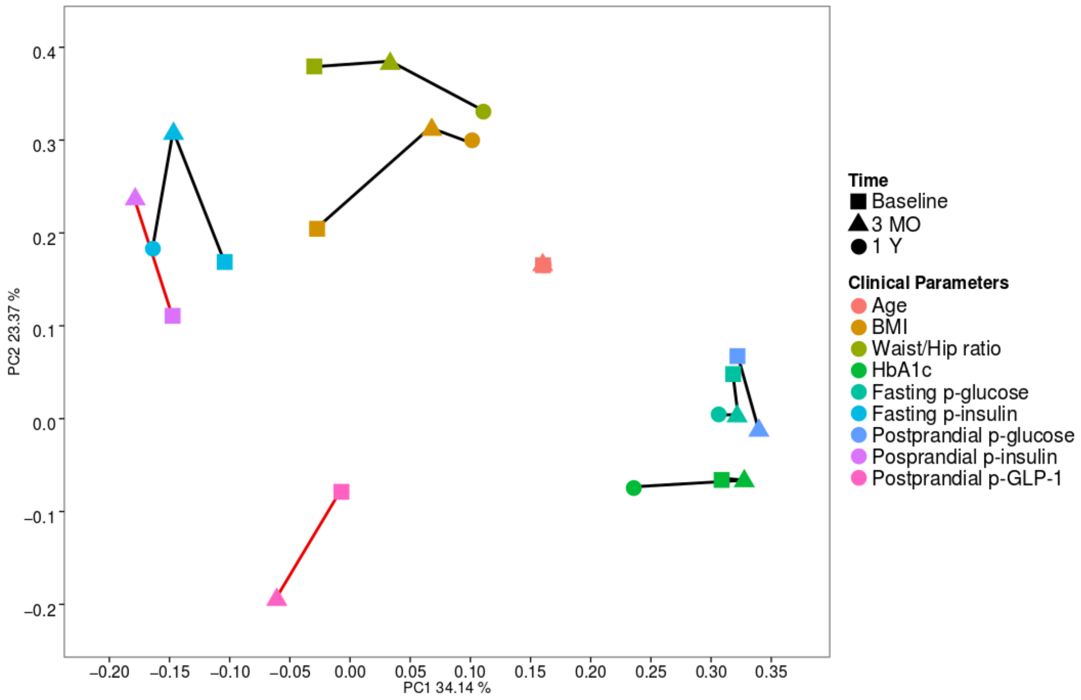

Figure S2. PCA of clinical parameters. Changes in the clinical markers at baseline, 3 months and 1 year after RYGB projected on the first two components of PCA. Measures were standard normalized to be comparable on relation to each other on the PCA space. While red arrows denote increase, black arrows denote decrease. Variance explained by each principal component is indicated next to PC 1 and 2 labels. HbA1c - glycated hemoglobin.

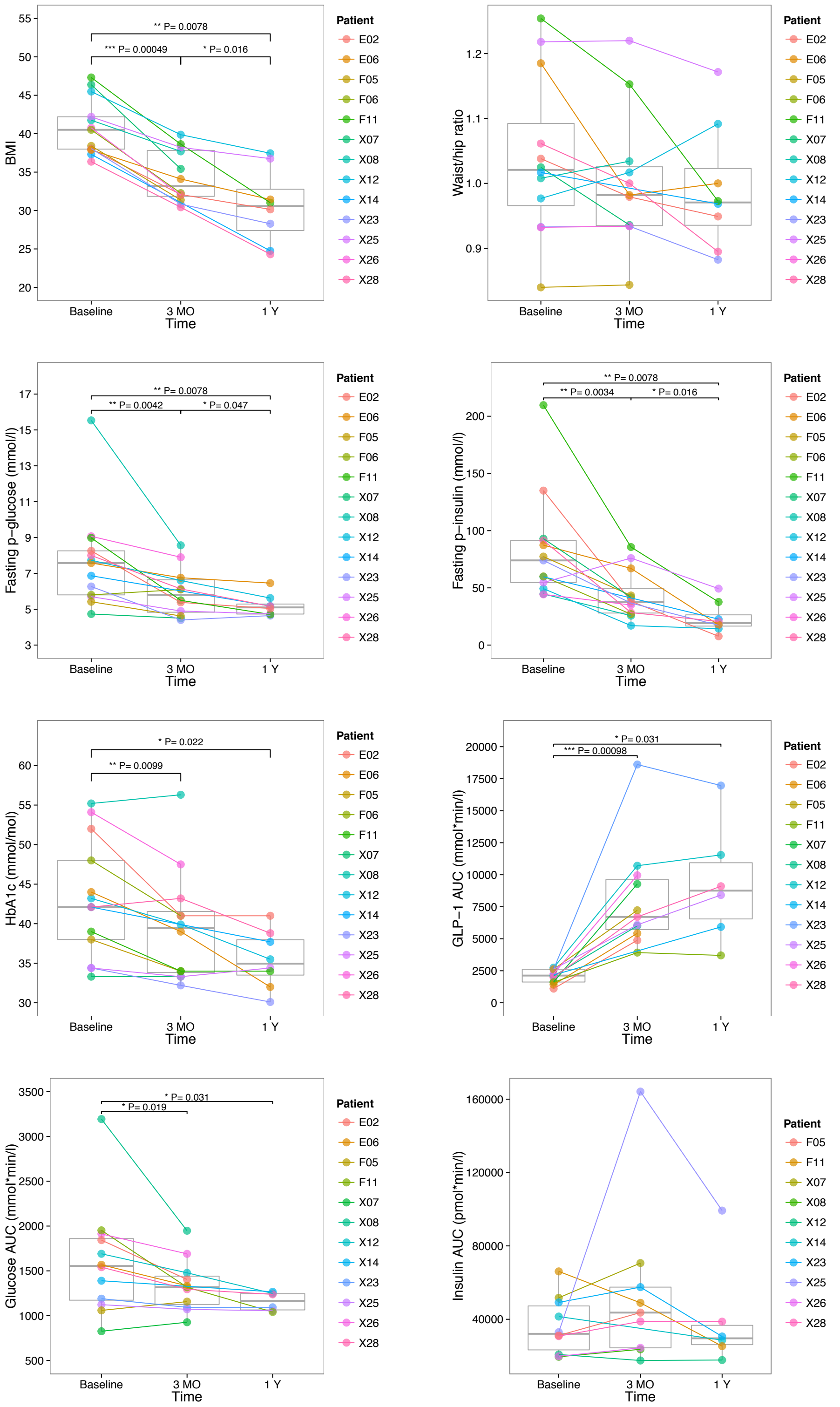

Figure S3. Clinical marker changes over time. Measures of the clinical parameters considered in the study over the time. Lines connect the measures from the same patient. We tested whether the clinical markers changes between the 3 time points were statistically significant using Wilcoxon signed-rank tests. P-values < 0.05 are denoted on top of the plots.

A)

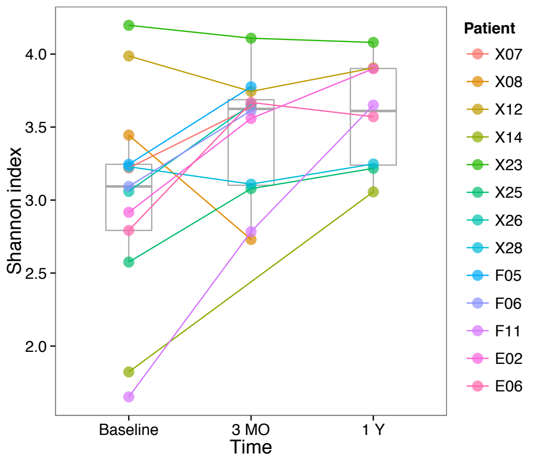

B)

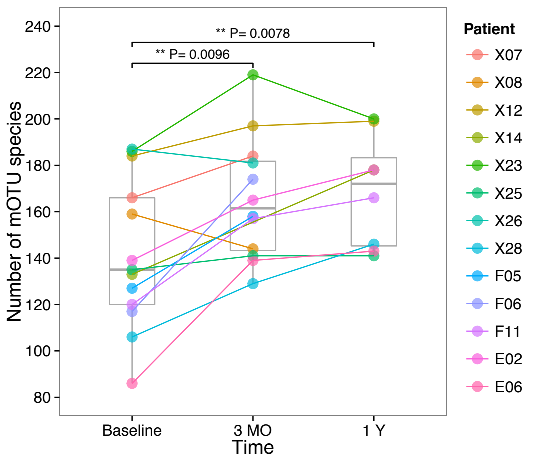

C)

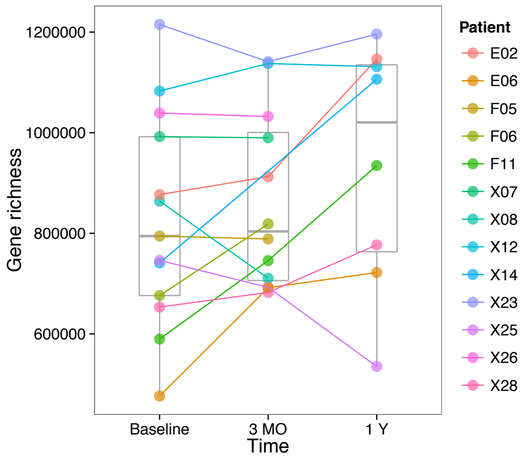

Figure S4. Microbial diversity over time. Diversity variation across samples over time represented as **A)** Shannon index **B)** number of mOTU species (richness), and **C)** gene richness. We tested by Wilcoxon signed-rank test whether there is significant difference in diversity between time points. P-values are denoted on top of the plots only when diversity changes were statistically significant ($P < 0.05$).

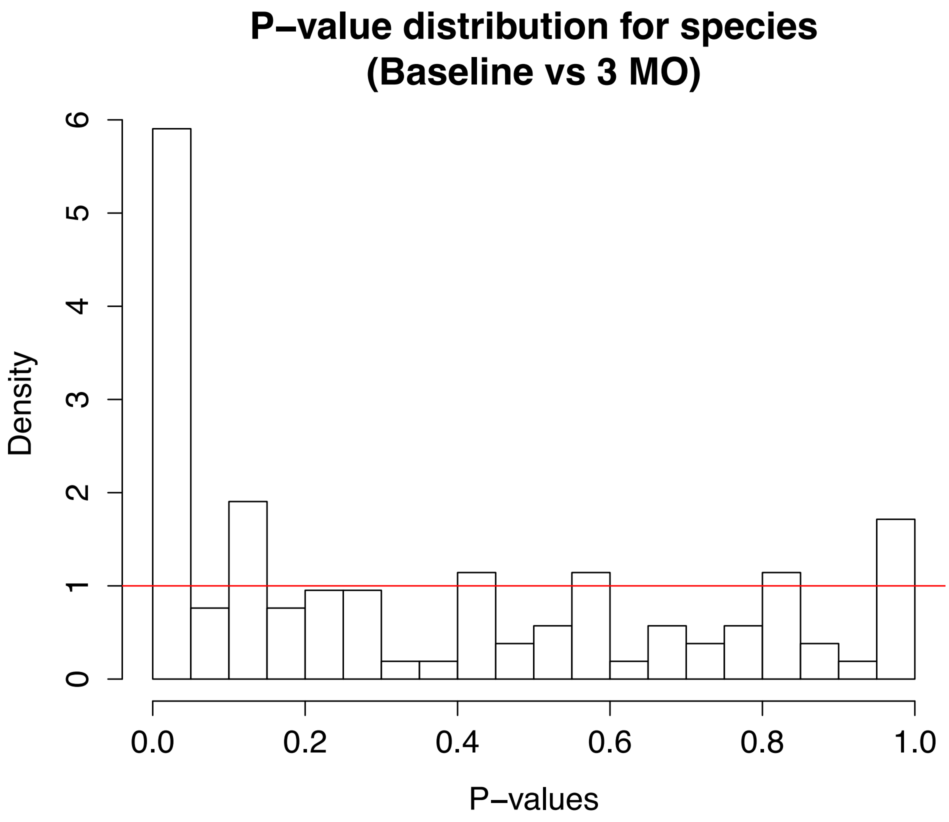

Figure S5. P-value distribution for mOTU species between baseline and 3 MO after RYGB. Histogram of the P-values obtained from the Wilcoxon signed-rank test when comparing mOTU species relative abundances between baseline and 3 MO after RYGB. The red line shows the estimated proportion of true null hypotheses. The estimated FDR was 17%.

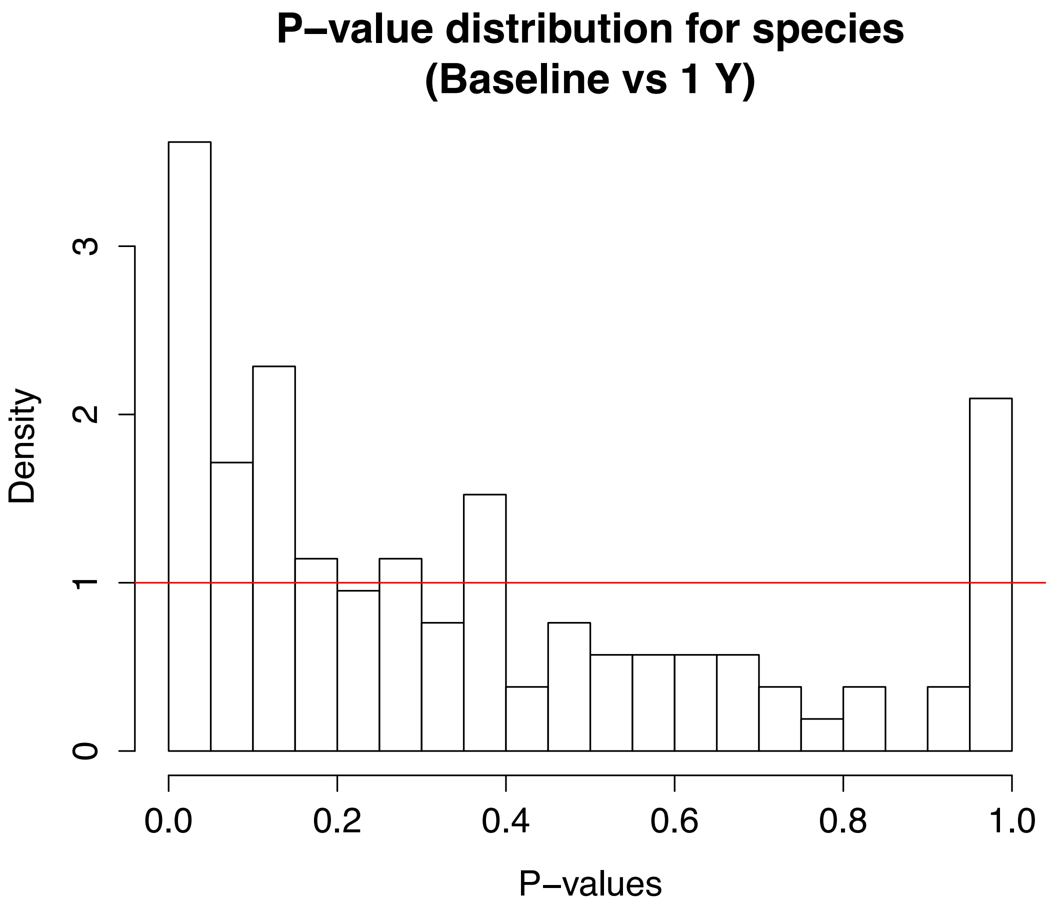

Figure S6. P-value distribution for mOTU species between baseline and 1 Y after RYGB. Histogram of the P-values obtained from the Wilcoxon signed-rank test when comparing mOTU species relative abundances between baseline and 1Y after RYGB. The red line shows the estimated proportion of true null hypotheses. The estimated FDR was 28%.

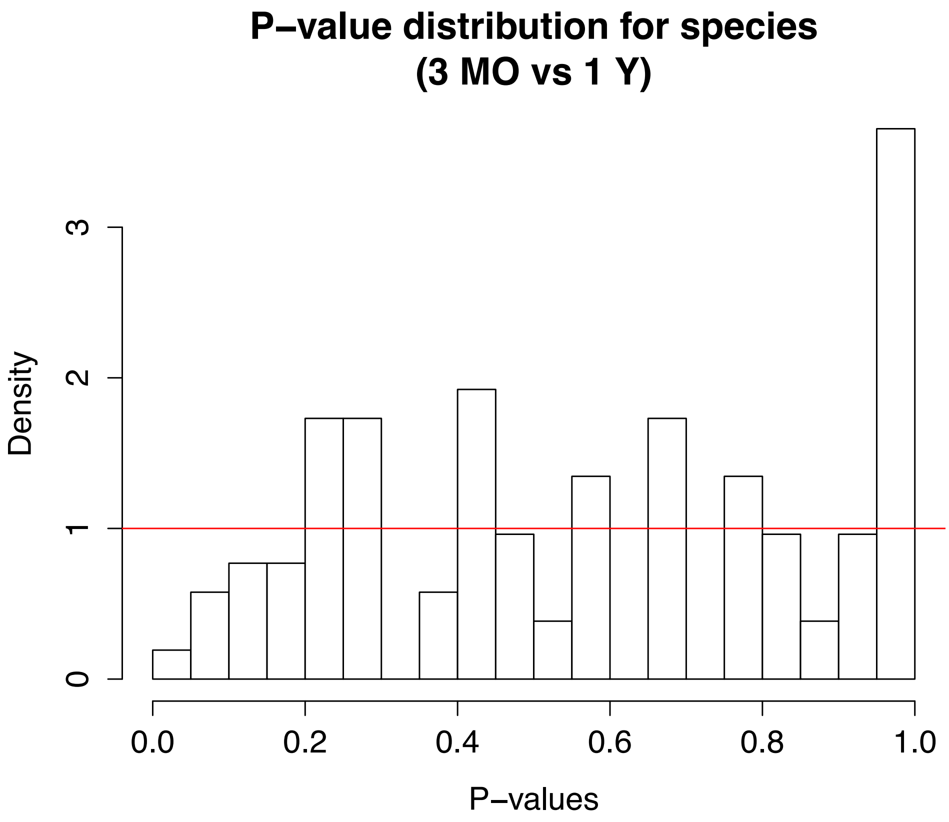

Figure S7. P-value distribution for mOTU species between 3 MO and 1 Y after RYGB. Histogram of the P-values obtained from the Wilcoxon signed-rank test when comparing mOTU species relative abundances between 3 MO and 1 Y after RYGB. The red line shows the estimated proportion of true null hypotheses. All significant species ($P < 0.05$) are clearly below this proportion.

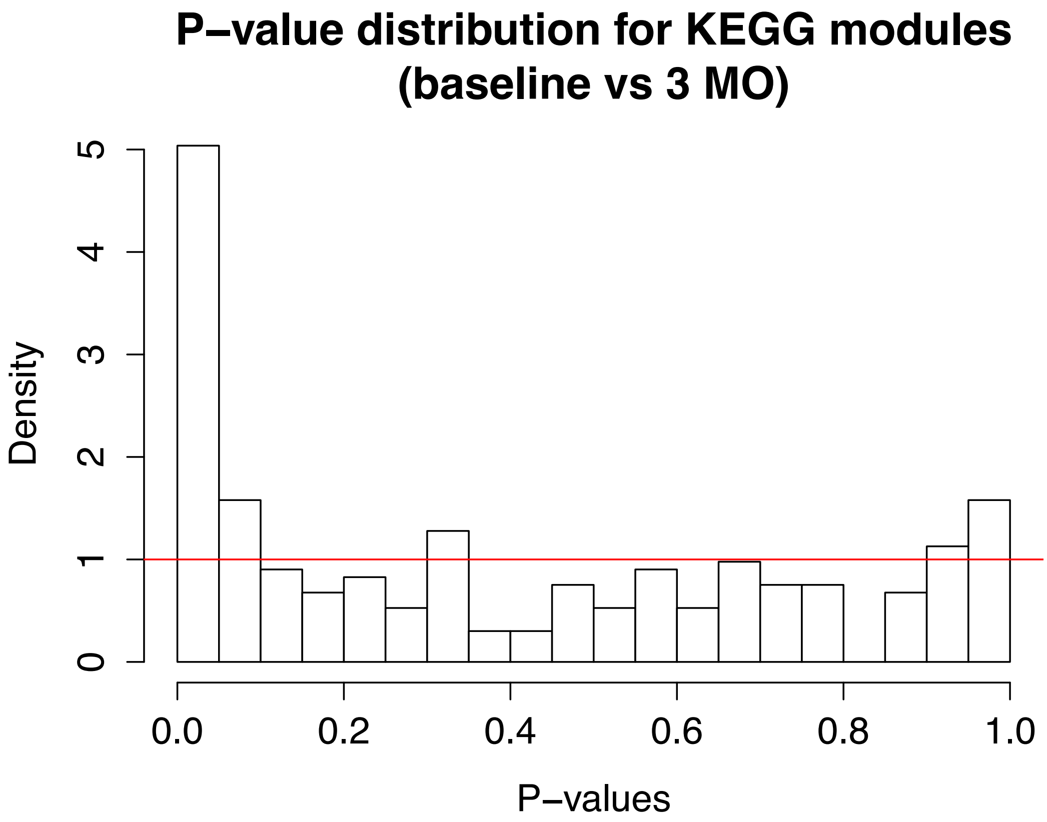

Figure S8. P-value distribution for KEGG modules between baseline and 3 MO after RYGB. Histogram of the P-values obtained from the Wilcoxon signed-rank test when comparing KEGG module relative abundances between baseline and 3 MO after RYGB. The red line shows the estimated proportion of true null hypotheses. The estimated FDR was 20%.

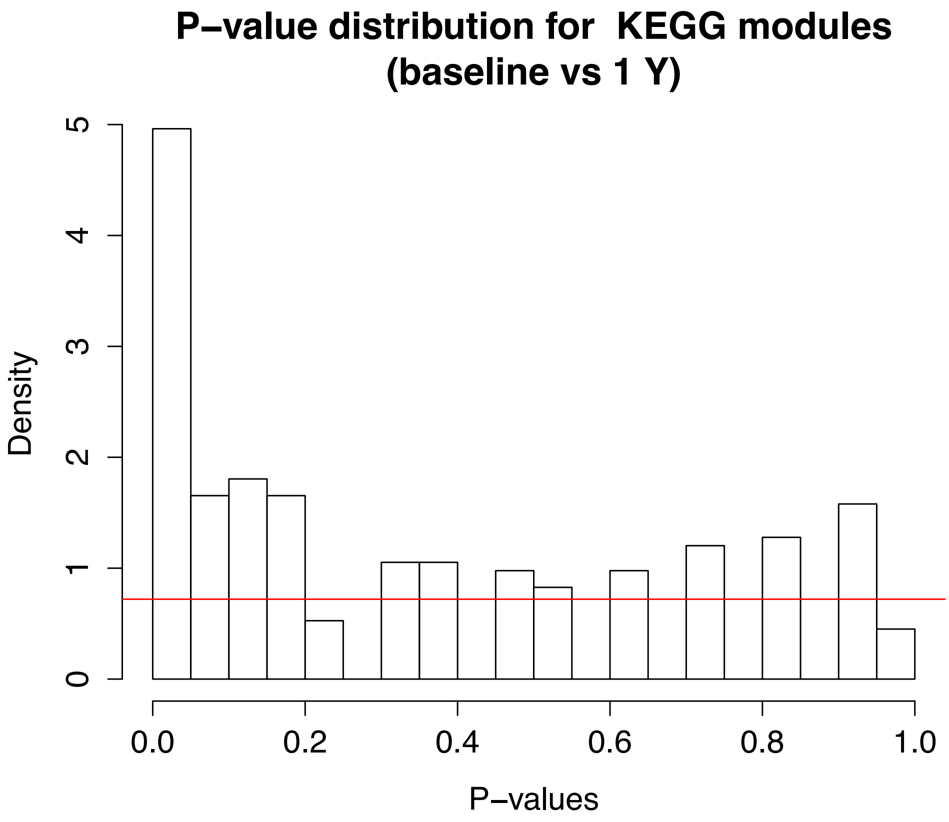

Figure S9. P-value distribution for KEGG modules between baseline and 1 Y after RYGB. Histogram of the P-values obtained from the Wilcoxon signed-rank test when comparing KEGG module relative abundances between baseline and 1 Y after RYGB. The red line shows the estimated proportion of true null hypotheses. The estimated FDR was 14%.

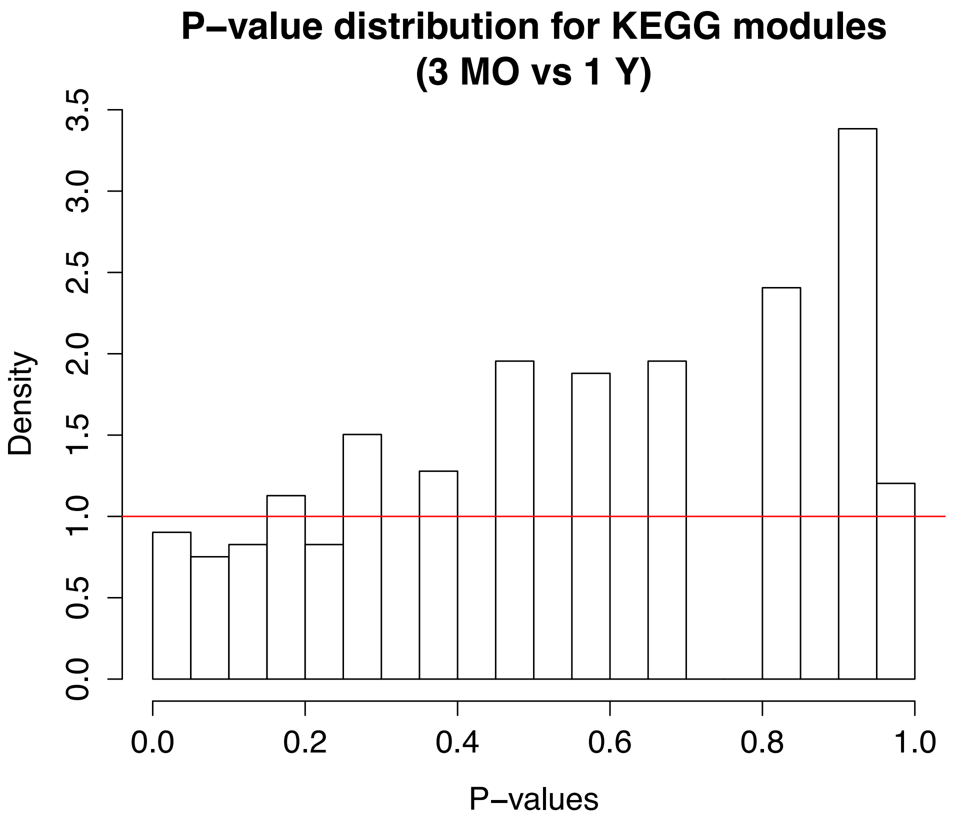

Figure S10. P-value distribution for KEGG modules between 3 MO and 1Y after RYGB. Histogram of the P-values obtained from the Wilcoxon signed-rank test when comparing KEGG module relative abundances between 3 MO and 1Y after RYGB. The red line shows the estimated proportion of true null hypotheses. All significant KEGG modules ($P < 0.05$) are clearly below this proportion.
